# Supplementary material for: Loop-Mediated Isothermal Amplification for the Fast Detection of Bonamia ostreae and Bonamia exitiosa in Flat Oysters
Source: Pathogens. 2024 Jan 30;13(2):132. doi: 10.3390/pathogens13020132 (PMC10893247; doi:10.3390/pathogens13020132)
Supplement: Supplementary file 1 [file pathogens-13-00132-s001.zip › Figure S1.pdf]

Figure S1. Nucleotide alignment of partial gene sequences of the *actin* and *18S rRNA* genes of bonamia species, their oyster hosts, and other haplosporidian sequences available in GenBank (accessed in March 2022). The LAMP primers binding sites are highlighted as follows: outer primers F3 and B3 coloured in red; inner primers FIP (F1c + F2) and BIP (B1c + B2) boxed in yellow and green; and loop primers LoopF and LoopB boxed in red.

A) Bonamia exitiosa actin gene alignment.

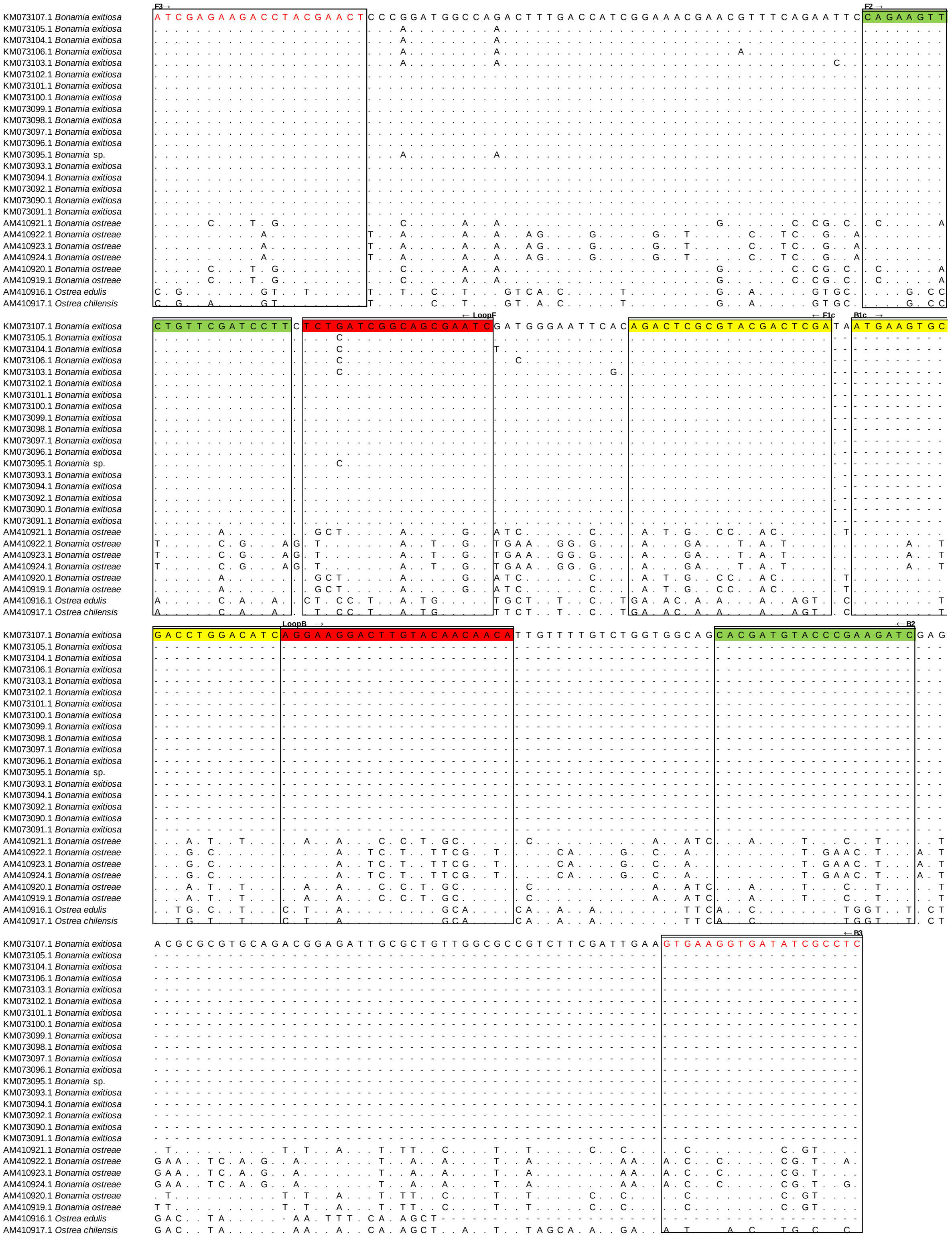

b) *Bonamia ostreae actin* 1 gene alignment.

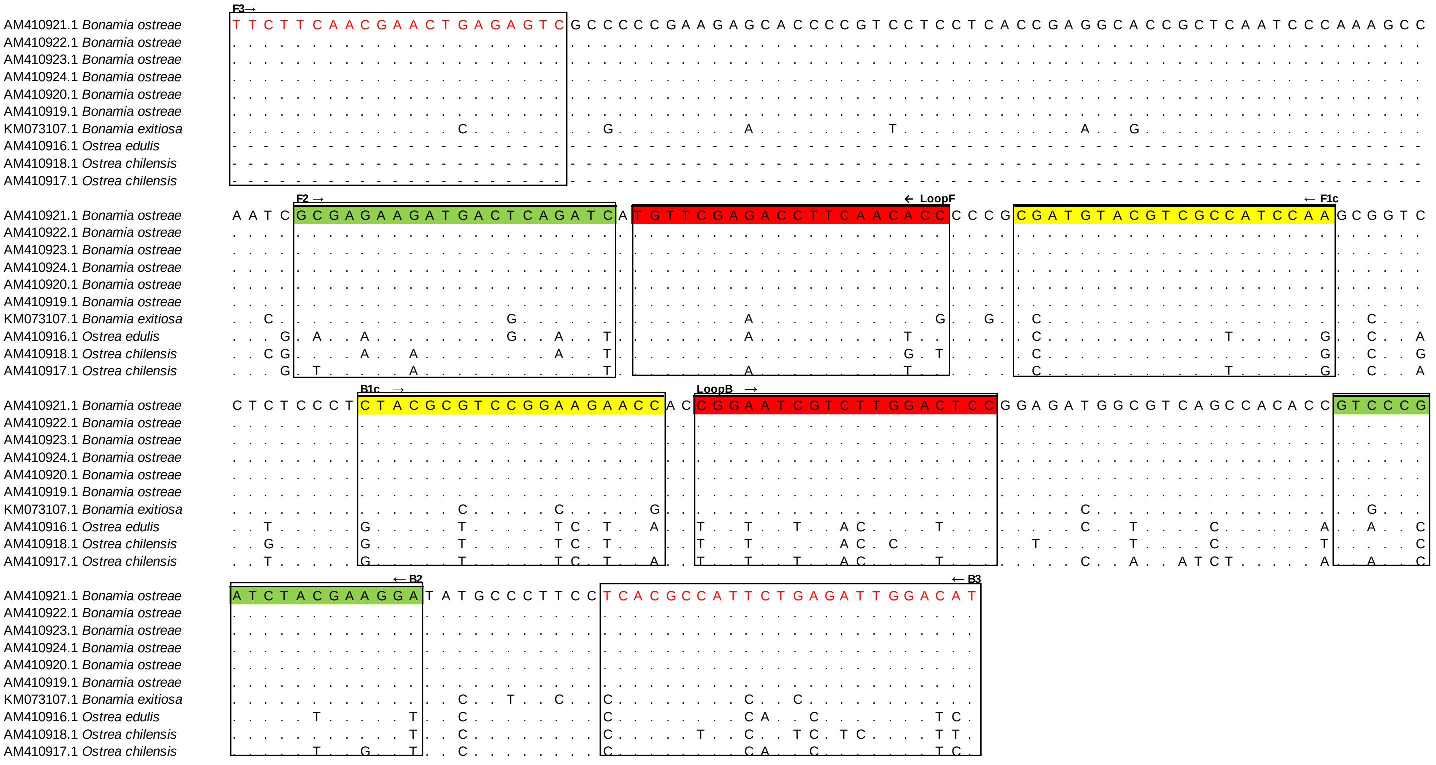

c) *Bonamia 18S rRNA* gene alignment.

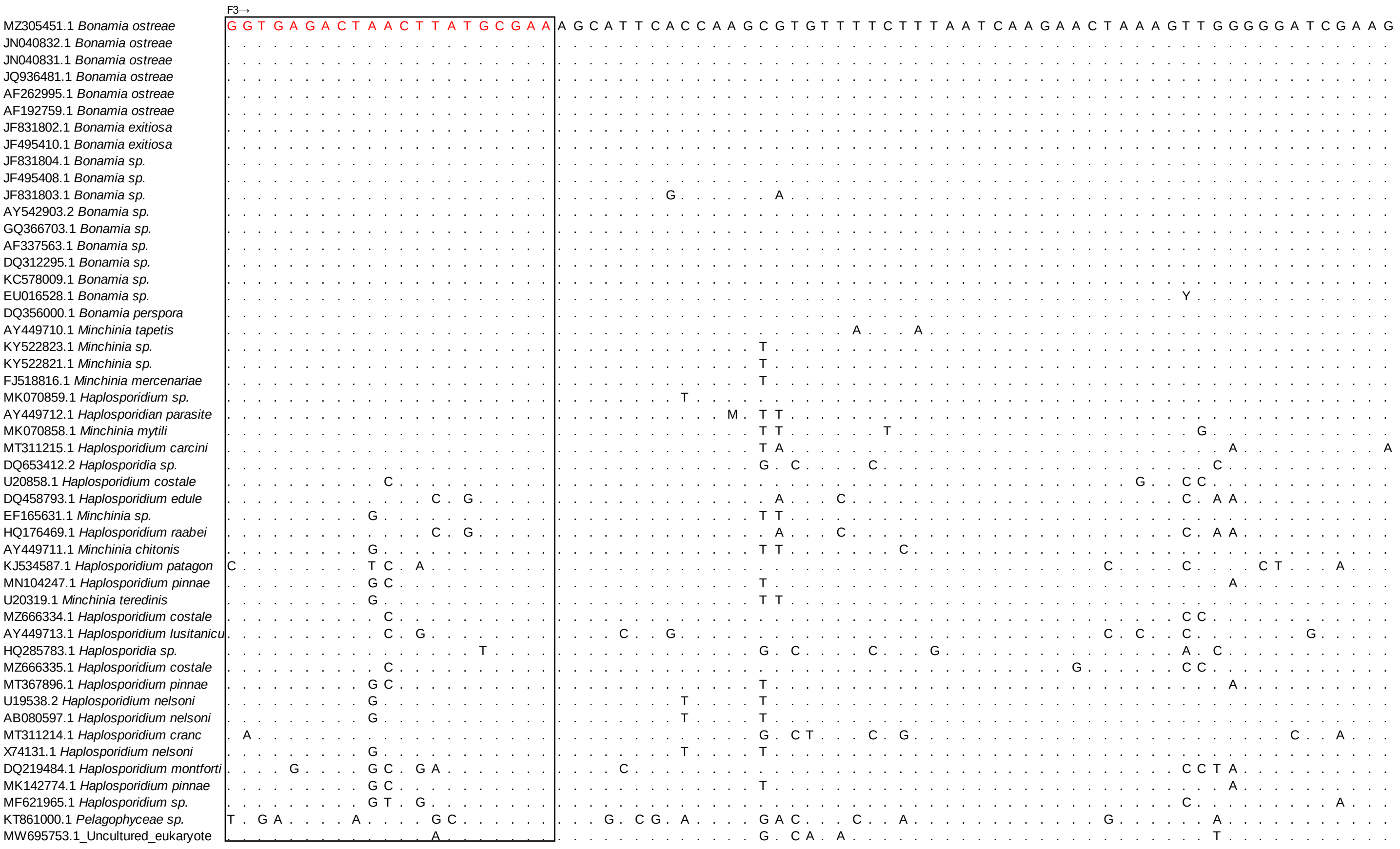

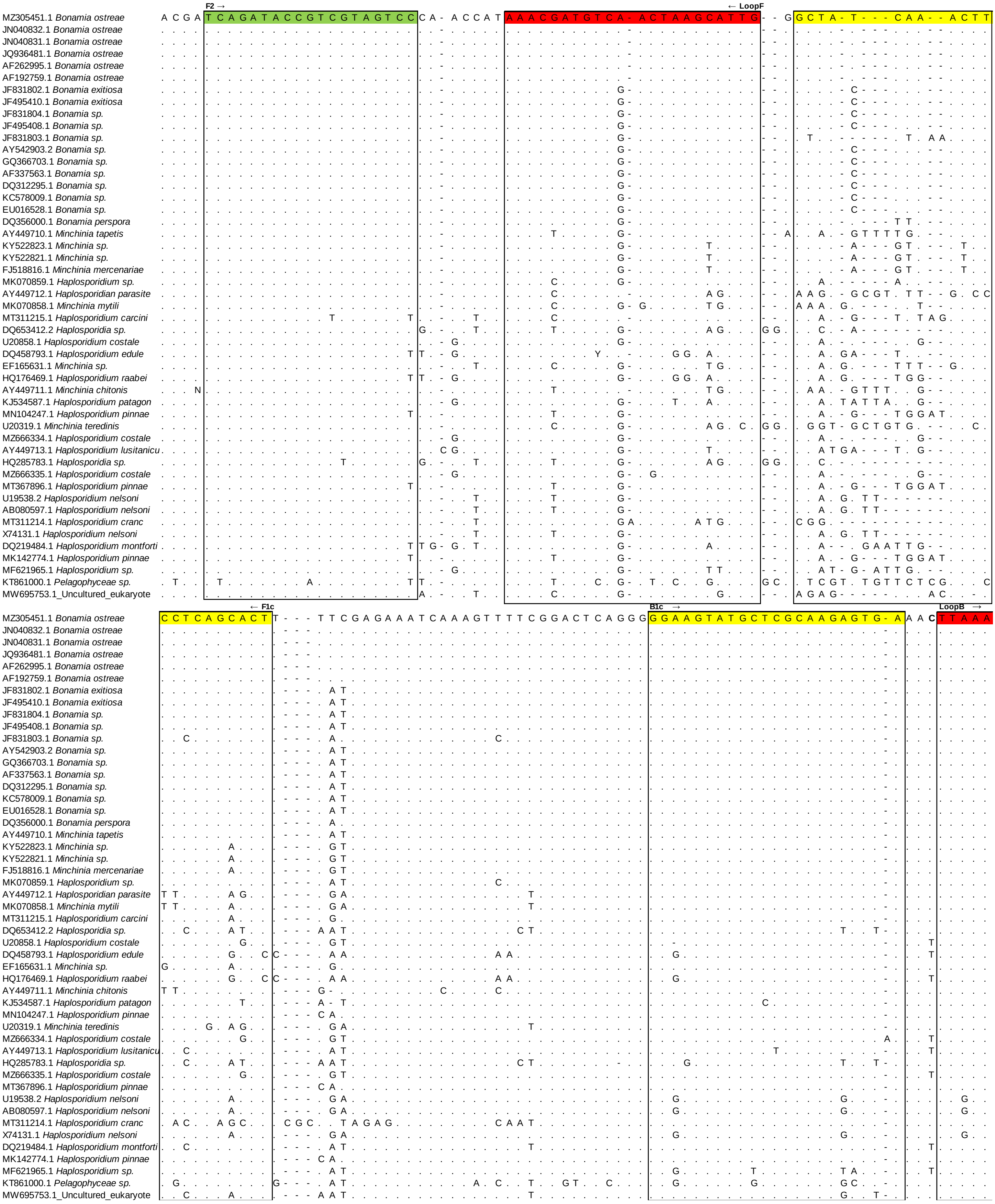
F2 →
← LoopF
← F1c
B1c →
LoopB →
MZ305451.1 Bonamia ostreae
JN040832.1 Bonamia ostreae
JN040831.1 Bonamia ostreae
JQ936481.1 Bonamia ostreae
AF262995.1 Bonamia ostreae
AF192759.1 Bonamia ostreae
JF831802.1 Bonamia exitiosa
JF495410.1 Bonamia exitiosa
JF831804.1 Bonamia sp.
JF495408.1 Bonamia sp.
JF831803.1 Bonamia sp.
AY542903.2 Bonamia sp.
GQ366703.1 Bonamia sp.
AF337563.1 Bonamia sp.
DQ312295.1 Bonamia sp.
KC578009.1 Bonamia sp.
EU016528.1 Bonamia sp.
DQ356000.1 Bonamia perspora
AY449710.1 Minchinia tapetis
KY522823.1 Minchinia sp.
KY522821.1 Minchinia sp.
FJ518816.1 Minchinia mercenariae
MK070859.1 Haplosporidium sp.
AY449712.1 Haplosporidian parasite
MK070858.1 Minchinia mytili
MT311215.1 Haplosporidium carcini
DQ653412.2 Haplosporidia sp.
U20858.1 Haplosporidium costale
DQ458793.1 Haplosporidium edule
EF165631.1 Minchinia sp.
HQ176469.1 Haplosporidium raabei
AY449711.1 Minchinia chitonis
KJ534587.1 Haplosporidium patagon
MN104247.1 Haplosporidium pinnae
U20319.1 Minchinia teredinis
MZ666334.1 Haplosporidium costale
AY449713.1 Haplosporidium lusitanicu
HQ285783.1 Haplosporidia sp.
MZ666335.1 Haplosporidium costale
MT367896.1 Haplosporidium pinnae
U19538.2 Haplosporidium nelsoni
AB080597.1 Haplosporidium nelsoni
MT311214.1 Haplosporidium cranc
X74131.1 Haplosporidium nelsoni
DQ219484.1 Haplosporidium montforti
MK142774.1 Haplosporidium pinnae
MF621965.1 Haplosporidium sp.
KT861000.1 Pelagophyceae sp.
MW695753.1_Uncultured_eukaryote

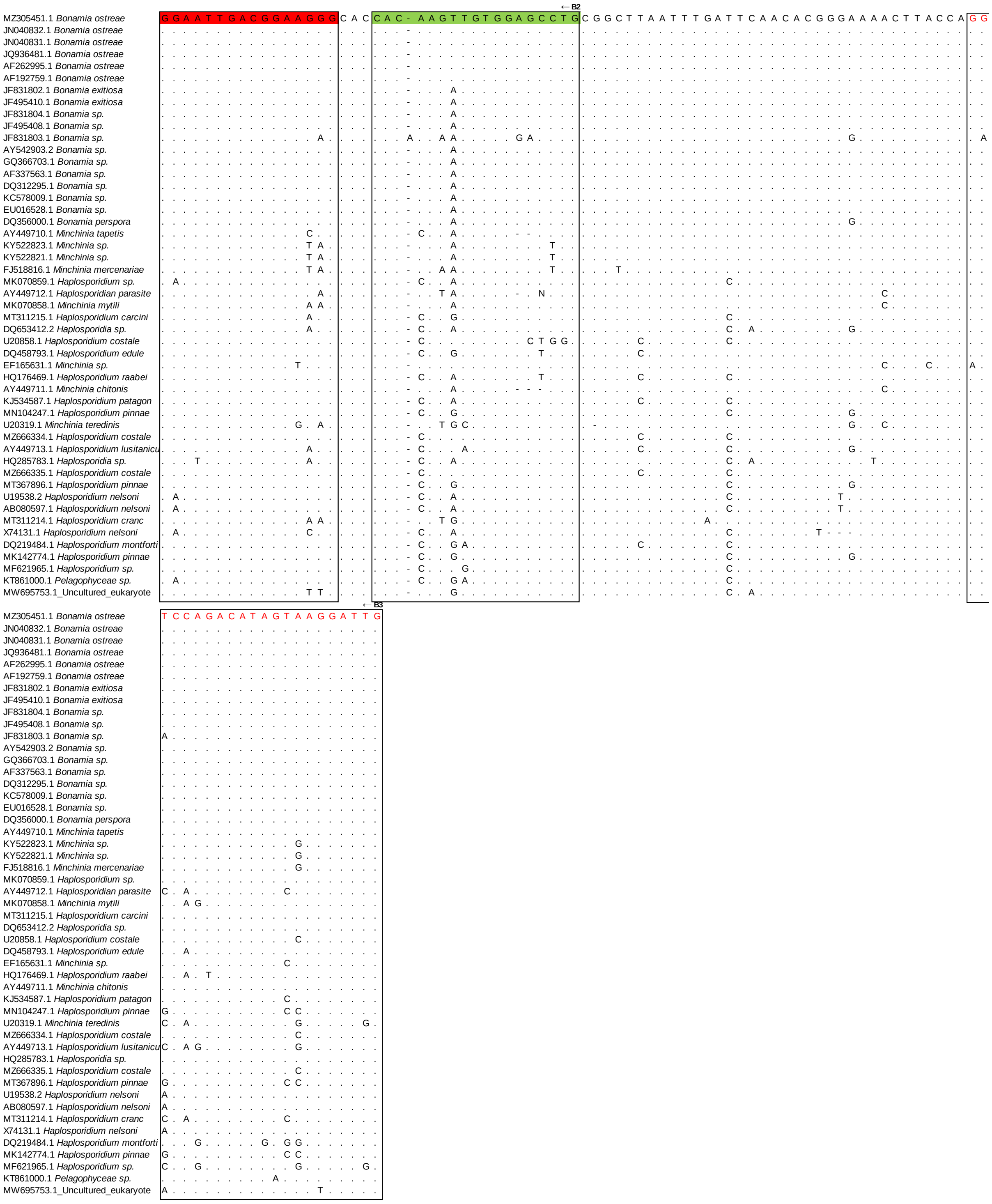
B2
B3
GGAATTGACGGAAGGGCACCAC-AAGTTGTGGAGCCTGCGGCTTAATTTGATTCAACACGGGAAAACTTACCAGG
TCCAGACATAGTAAGGATTG
MZ305451.1 Bonamia ostreae
JN040832.1 Bonamia ostreae
JN040831.1 Bonamia ostreae
JQ936481.1 Bonamia ostreae
AF262995.1 Bonamia ostreae
AF192759.1 Bonamia ostreae
JF831802.1 Bonamia exitiosa
JF495410.1 Bonamia exitiosa
JF831804.1 Bonamia sp.
JF495408.1 Bonamia sp.
JF831803.1 Bonamia sp.
AY542903.2 Bonamia sp.
GQ366703.1 Bonamia sp.
AF337563.1 Bonamia sp.
DQ312295.1 Bonamia sp.
KC578009.1 Bonamia sp.
EU016528.1 Bonamia sp.
DQ356000.1 Bonamia perspora
AY449710.1 Minchinia tapetis
KY522823.1 Minchinia sp.
KY522821.1 Minchinia sp.
FJ518816.1 Minchinia mercenariae
MK070859.1 Haplosporidium sp.
AY449712.1 Haplosporidian parasite
MK070858.1 Minchinia mytili
MT311215.1 Haplosporidium carcini
DQ653412.2 Haplosporidia sp.
U20858.1 Haplosporidium costale
DQ458793.1 Haplosporidium edule
EF165631.1 Minchinia sp.
HQ176469.1 Haplosporidium raabei
AY449711.1 Minchinia chitonis
KJ534587.1 Haplosporidium patagon
MN104247.1 Haplosporidium pinnae
U20319.1 Minchinia teredinis
MZ666334.1 Haplosporidium costale
AY449713.1 Haplosporidium lusitanicu
HQ285783.1 Haplosporidia sp.
MZ666335.1 Haplosporidium costale
MT367896.1 Haplosporidium pinnae
U19538.2 Haplosporidium nelsoni
AB080597.1 Haplosporidium nelsoni
MT311214.1 Haplosporidium cranc
X74131.1 Haplosporidium nelsoni
DQ219484.1 Haplosporidium montforti
MK142774.1 Haplosporidium pinnae
MF621965.1 Haplosporidium sp.
KT861000.1 Pelagophyceae sp.
MW695753.1_Uncultured_eukaryote
