# Supplementary material for: Loop-Mediated Isothermal Amplification for the Fast Detection of Bonamia ostreae and Bonamia exitiosa in Flat Oysters
Source: Pathogens. 2024 Jan 30;13(2):132. doi: 10.3390/pathogens13020132 (PMC10893247; doi:10.3390/pathogens13020132)

Figure S2. Example of positivity tests on a Genie instrument to determine the product annealing curve temperature range for the *Bonamia ostreae actin 1* LAMP assay. a) Isothermal amplification of a range of infected and uninfected samples of *Ostrea edulis*. b) Anneal derivative of the isothermal amplified products showing the peaks in the 89.68 ± 013 °C range. c) Electropherogram profile comparison of the samples included in the test analysed in a TapeStation. d) Gel image profile comparison in a TapeStation. Positive samples showed a high molecular band smear pattern. Ladder from 100 to 48500 bp. Positive samples: BO, VO, G3, 5M, 54, and D5; negative samples: VB.

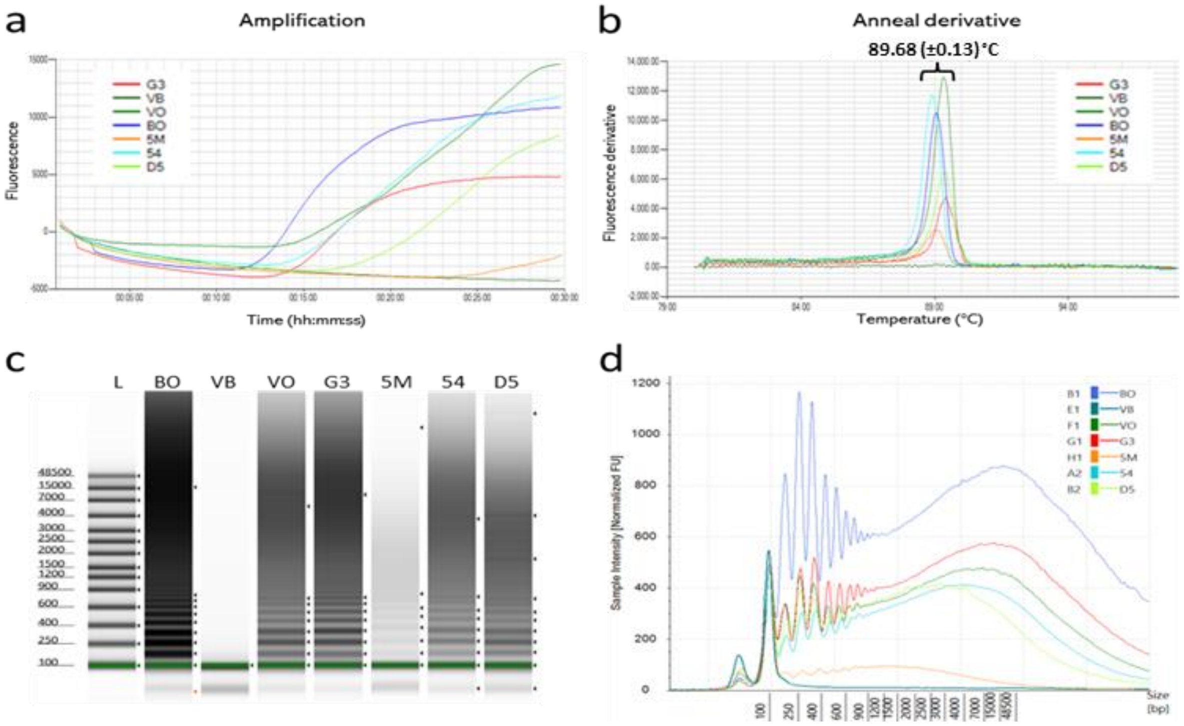

Supplement: Supplementary file 1 [file pathogens-13-00132-s001.zip › Figure S2.pdf]
