# Supplementary material for: Loop-Mediated Isothermal Amplification for the Fast Detection of Bonamia ostreae and Bonamia exitiosa in Flat Oysters
Source: Pathogens. 2024 Jan 30;13(2):132. doi: 10.3390/pathogens13020132 (PMC10893247; doi:10.3390/pathogens13020132)

Figure S3. Example of specificity tests of (A) *Bonamia ostreae actin* 1, (B) *B. exitiosa actin*, and (C) *Bonamia sp. 18S* LAMP assays on a Genie instrument. Each test was conducted with positive samples of *B. ostreae* (Bo), *B. exitiosa* (Be), and uninfected tissues of *Ostrea edulis* (Oe) and *Crassostrea gigas* (Cg). The isothermal amplification, the LAMP product anneal temperature, the electropherogram profile, and the gel image in a TapeStation are shown for each LAMP assay. Positive samples showed a high molecular band smear pattern corresponding to the amplification and concatenation of the LAMP product.

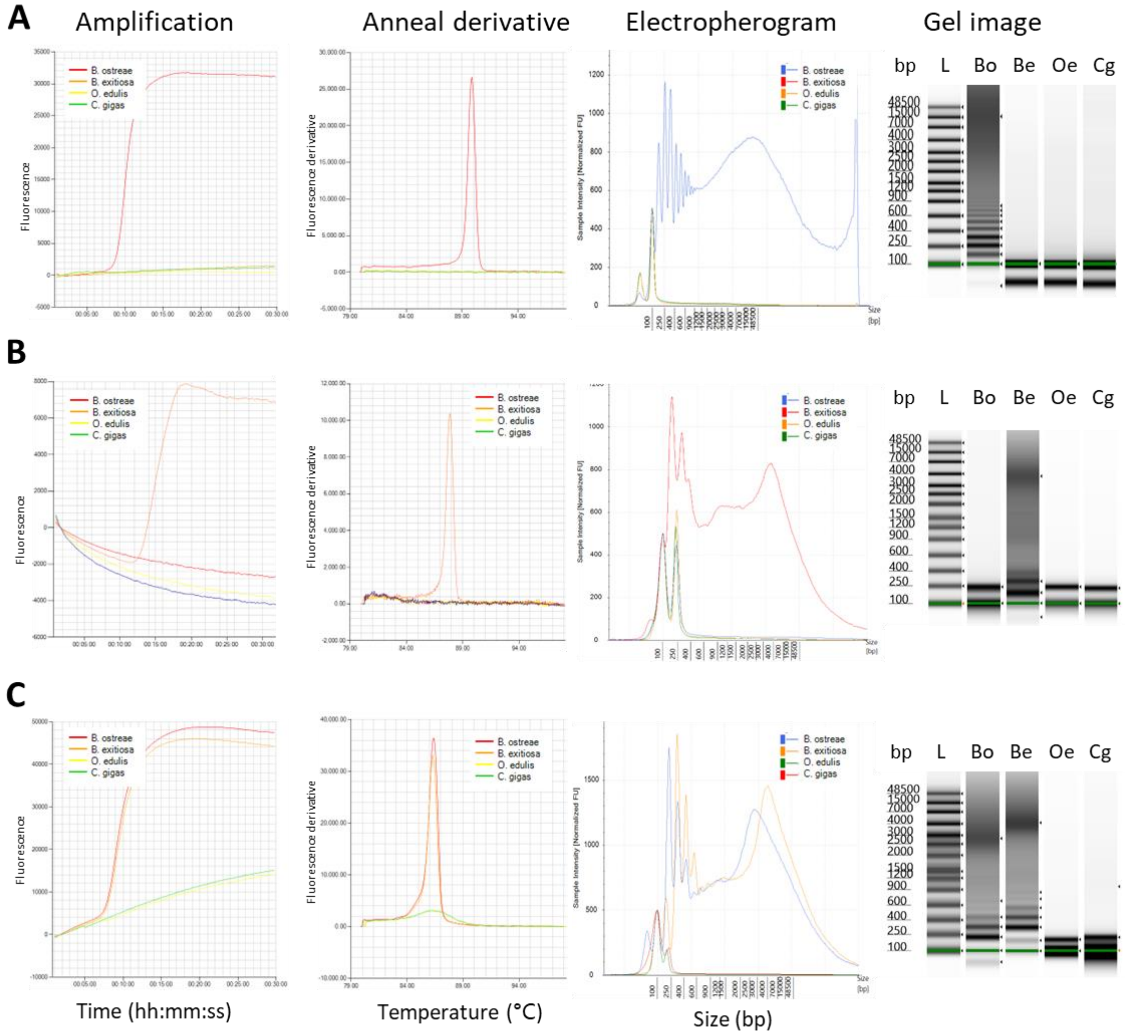

Supplement: Supplementary file 1 [file pathogens-13-00132-s001.zip › Figure S3.pdf]
