# Supplementary material for: Loop-Mediated Isothermal Amplification for the Fast Detection of Bonamia ostreae and Bonamia exitiosa in Flat Oysters
Source: Pathogens. 2024 Jan 30;13(2):132. doi: 10.3390/pathogens13020132 (PMC10893247; doi:10.3390/pathogens13020132)

Figure S4. Accuracy tests. Loop-mediated isothermal amplification of *Bonamia ostreae* and *B. exitiosa* gDNA using either the species-specific actin LAMP assays or the generic *Bonamia sp*. 18S LAMP assay in a Genie instrument. The time of positivity (Tp), amplification, and anneal derivative are shown for each LAMP test. NA: no amplification.

A) *Bonamia ostreae actin* LAMP assay

B. ostreae actin **LAMP assay. Samples 1-6. Run GEN3-1235_0065**

Well	Sample	Tp (min:sec)	Anneal derivative (°C)
1	Positive control	14:15	89.7
2	1	12:00	89.6
3	2	na	na
4	3	na	na
5	4	na	na
6	5	na	na
7	6	12:00	89.5
8	Negative control	na	na

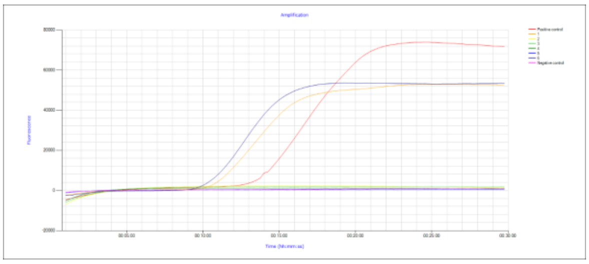

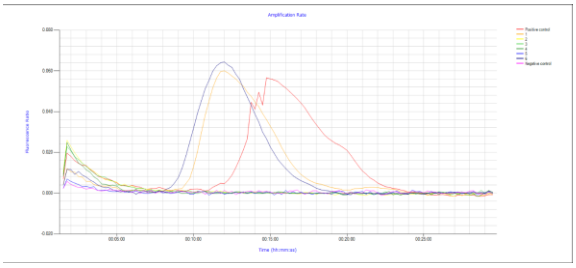

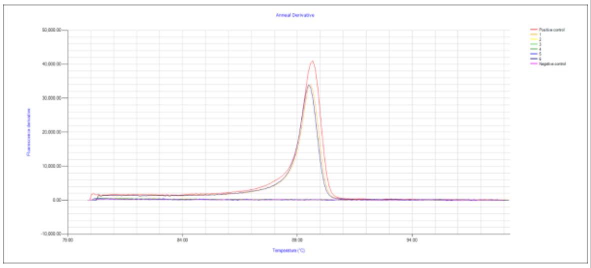

B. ostreae actin **LAMP assay. Samples 7-12. Run GEN3-1235_0068**

Well	Sample	Tp (min:sec)	Anneal derivative (°)
1	Positive control	10:30	89.9
2	7	na	na
3	8	na	na
4	9	na	na
5	10	08:30	89.7
6	11	na	na
7	12	na	na
8	Negative control	na	na

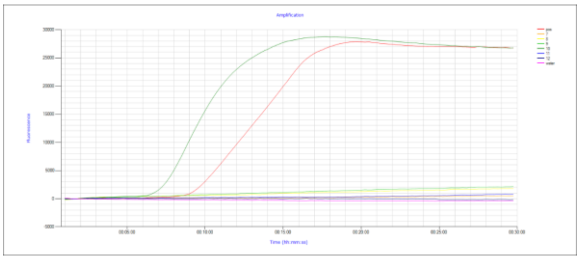

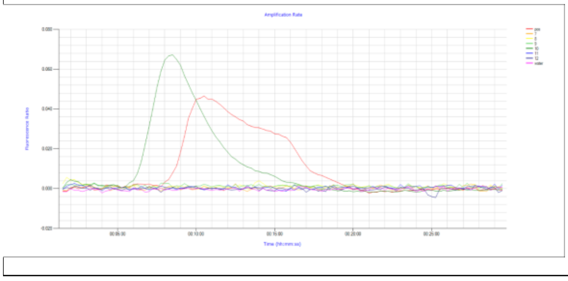

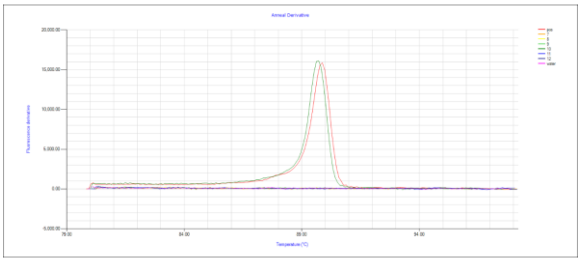

B. ostreae actin **LAMP assay. Samples 13-18. Run GEN3-1235_0063**

Well	Sample	Tp (min:sec)	Anneal derivative (°)
1	Positive control	10:30	89.8
2	13	na	na
3	14	16:45	89.5
4	15	09:00	89.7
5	16	08:30	89.6
6	17	na	na
7	18	09:00	89.6
8	Negative control	na	na

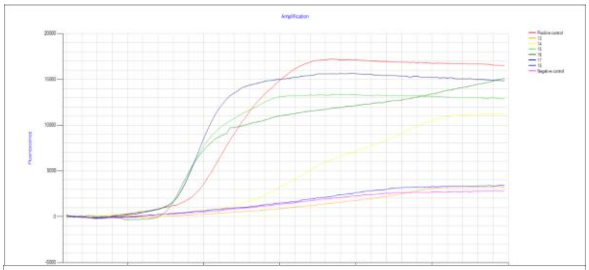

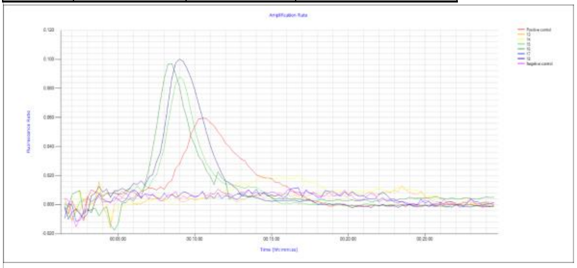

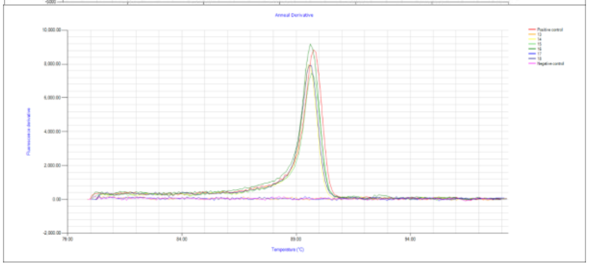

B. ostreae actin **LAMP assay. Samples 19-24. Run GEN3-1235_0064**

Well	Sample	Tp (min:sec)	Anneal derivative (°)
1	Positive control	16:00	89.9
2	19	07:45	89.7
3	20	na	na
4	21	na	na
5	22	na	na
6	23	08:15	89.8
7	24	09:00	89.6
8	Negative control	na	na

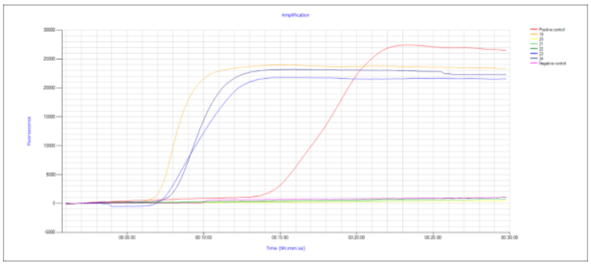

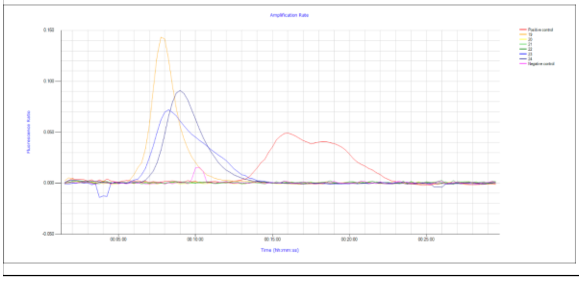

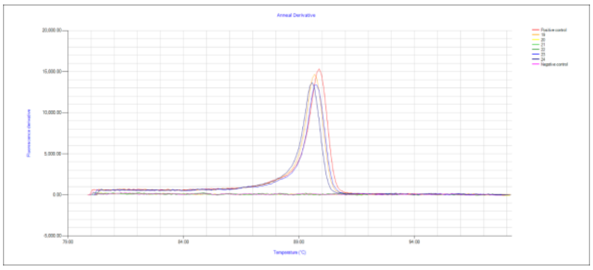

B) ***Bonamia exitiosa actin* LAMP assay.**

B. exitiosa actin **LAMP assay. Samples 1-6. Run GEN2-1167_0161 (A)**

Well	Sample	Tp (min:sec)	Anneal derivative (°C)
1	1	11:00	86.8
2	2	na	na
3	3	17:30	86.8
4	4	na	na
5	5	na	na
6	6	na	na
7	Positive control	09:15	87.1
8	Negative control	na	na

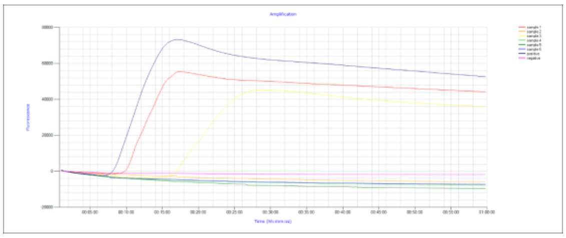

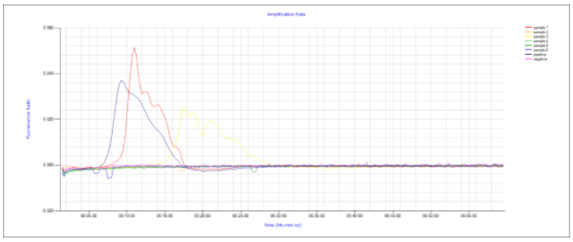

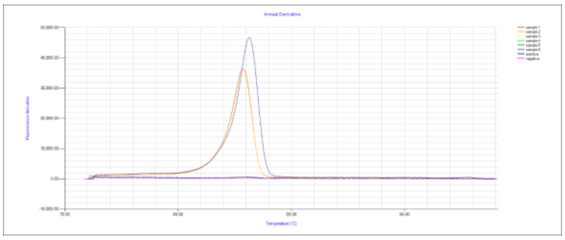

B. exitiosa actin **LAMP assay. Samples 7-12. Run GEN3-1235_0069**

Well	Sample	Tp (min:sec)	Anneal derivative (°C)
1	Positive control	13:45	87.2
2	7	na	na
3	8	12:45	87.3
4	9	17:30	87.3
5	10	na	na
6	11	15:15	87.3
7	12	na	na
8	Negative control	na	na

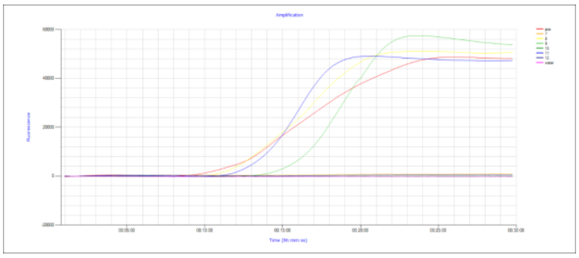

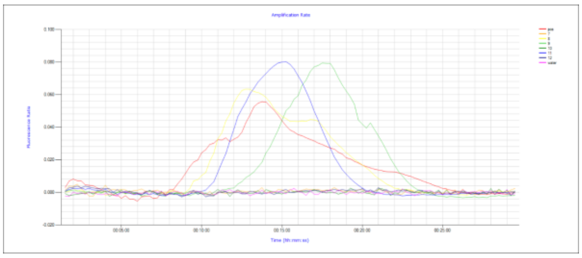

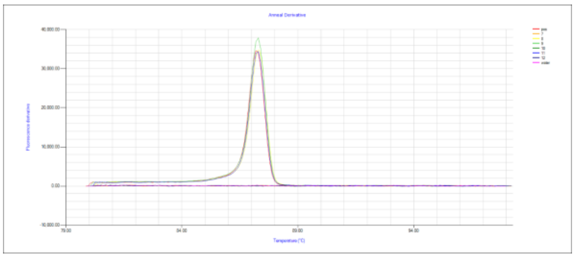

B. exitiosa actin **LAMP assay. Samples 13-18. Run GEN2-1167_0161 (B)**

Well	Sample	Tp (min:sec)	Anneal derivative (°C)
1	13	na	na
2	14	12:00	86.9
3	15	13:40	86.8
4	16	na	na
5	17	12:00	86.8
6	18	10:15	86.9
7	Positive control	12:00	87
8	Negative control	na	na

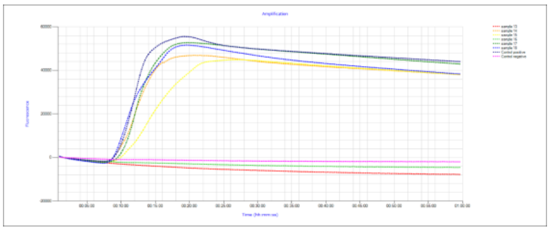

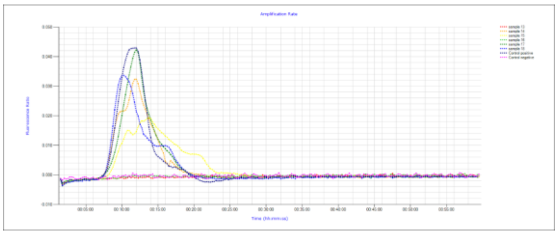

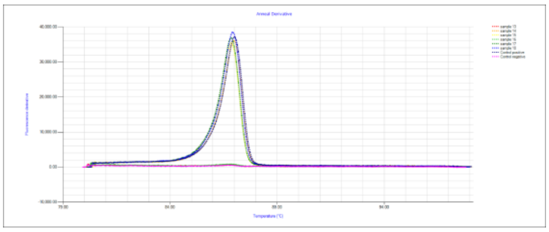

B. exitiosa actin **LAMP assay. Samples 19-24. Run GEN2-1167_0162**

Well	Sample	TP (min:sec)	Anneal derivative (°C)
1	19	na	87
2	20	na	na
3	21	na	na
4	22	na	na
5	23	na	na
6	24	na	na
7	Positive control	09:15	na
8	Negative control	na	na

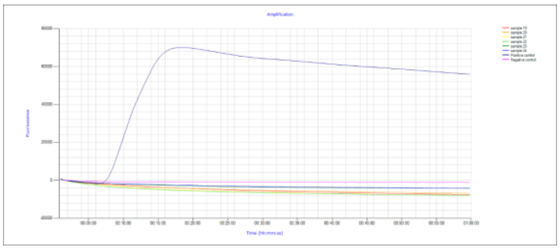

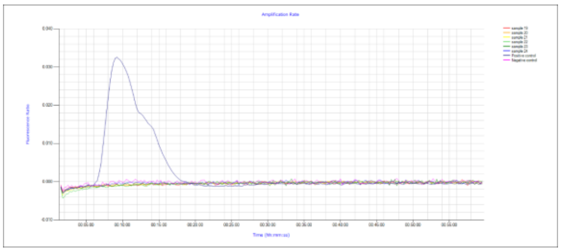

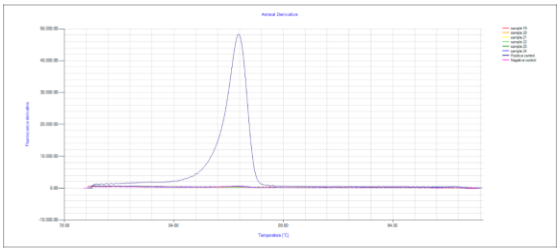

C) Generic Bonamia 18S LAMP assay.

Generic Bonamia 18S LAMP assay. Samples 1-6. Run GEN3-1235_0078

Well	Sample	Tp (min:sec)	Anneal derivative (°C)
1	Positive control	11:44	85.9
2	1	11:59	85.9
3	2	na	85.9
4	3	12:14	85.8
5	4	11:44	85.7
6	5	na	na
7	6	09:59	85.9
8	Negative control	na	na

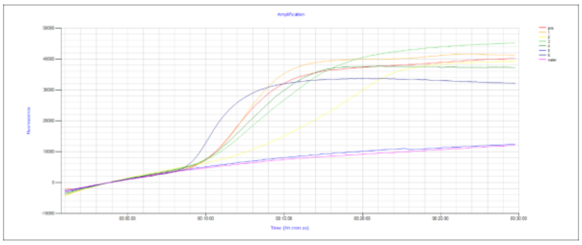

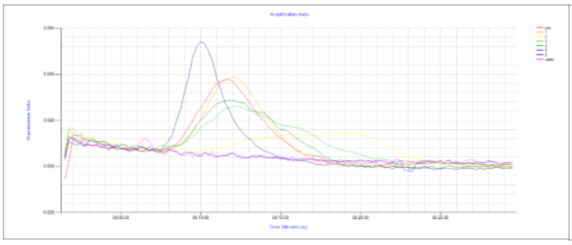

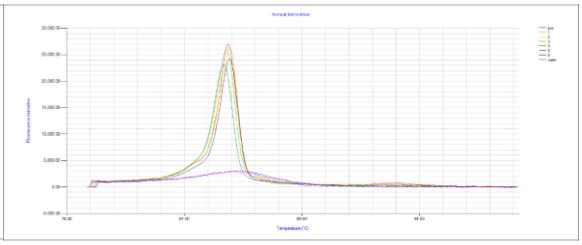

Generic Bonamia 18S LAMP assay. Samples 7-12. Run GEN3-1235_0075

Well	Sample	Tp (min:sec)	Anneal derivative (°C)
1	Positive control	10:15	85.9
2	7	na	na
3	8	13:15	86.1
4	9	12:30	86
5	10	09:15	86
6	11	10:00	86
7	12	na	na
8	Negative control	na	na

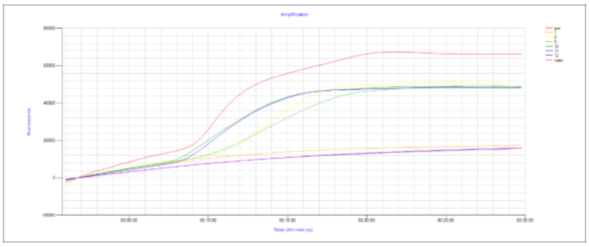

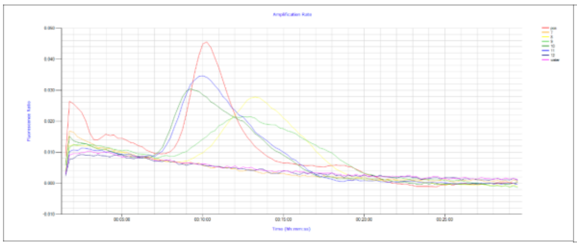

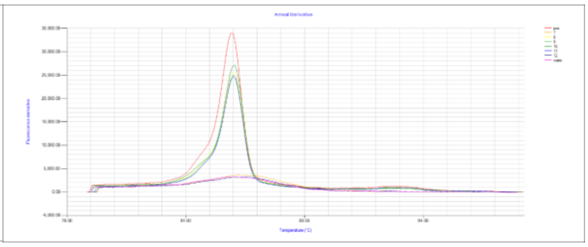

Generic Bonamia 18S LAMP assay. Samples 13-18. Run GEN3-1235_0076

Well	Sample	Tp (min:sec)	Anneal derivative (°C)
1	Positive control	10:15	86
2	13	na	na
3	14	10:15	86.2
4	15	10:00	86.1
5	16	10:30	85.9
6	17	09:30	85.9
7	18	13:00	85.8
8	Negative control	na	na

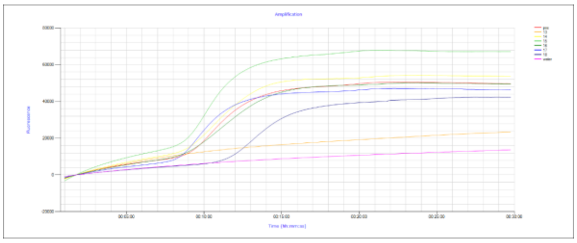

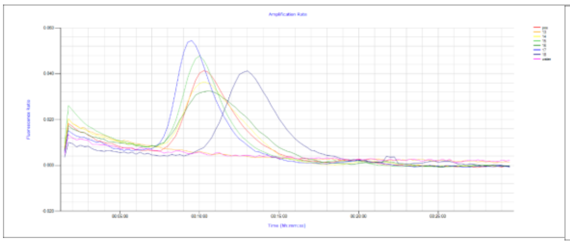

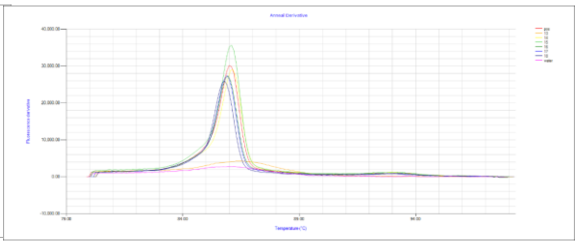

Generic Bonamia 18S LAMP assay. Samples 19-24. Run GEN3-1235_0077

Well	Sample	Tp (min:sec)	Anneal derivative (°C)
1	Positive control	09:14	85.9
2	19	09:29	86
3	20	na	na
4	21	na	na
5	22	na	na
6	23	10:14	86
7	24	09:14	85.9
8	Negative control	na	0

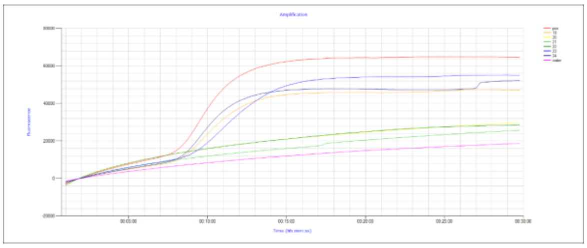

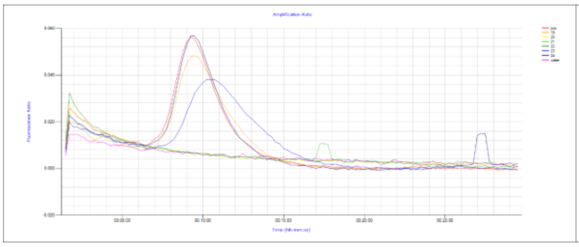

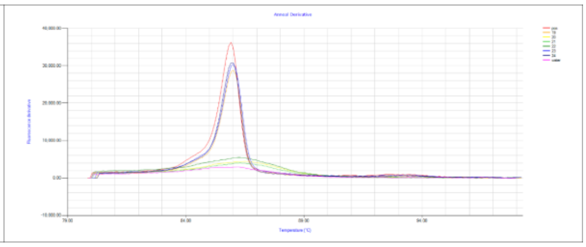

Supplement: Supplementary file 1 [file pathogens-13-00132-s001.zip › Figure S4.pdf]
