# Supplementary material for: Loop-Mediated Isothermal Amplification for the Fast Detection of Bonamia ostreae and Bonamia exitiosa in Flat Oysters
Source: Pathogens. 2024 Jan 30;13(2):132. doi: 10.3390/pathogens13020132 (PMC10893247; doi:10.3390/pathogens13020132)
Supplement: Supplementary file 1 [file pathogens-13-00132-s001.zip › Table S1.pdf]

Table S1. Set of 24 oyster gDNA samples used to assess the diagnostic accuracy of the three LAMP assays. The sample status: negative, positive (+), or strong positive (++), was established previously by Taqman PCR and conventional PCR.

Pool	Replicates	Sample status	Sample ID
1	1-1	Negative	**20**
	1-2	Negative	**21**
	1-3	Negative	**13**
2	2-1	Negative	**7**
	2-3	Negative	**2**
	2-3	Negative	**5**
3	3-1	Negative	**12**
	3-2	Negative	**22**
4	4-1	*B. exitiosa* ++	**17**
	4-2	*B. exitiosa* ++	**11**
	4-3	*B. exitiosa* ++	**4**
5	5-1	*B. exitiosa* +	**8**
	5-2	*B. exitiosa* +	**9**
	5-3	*B. exitiosa* +	**3**
6	6-1	*B. ostreae* ++	**6**
	6-2	*B. ostreae* ++	**24**
	6-3	*B. ostreae* ++	**19**
7	7-1	*B. ostreae* +	**23**
	7-2	*B. ostreae* +	**16**
	7-3	*B. ostreae* +	**10**
8	8-1	*B. ostreae* ++ & *B. exitiosa* +	**15**
	8-2	*B. ostreae* ++ & *B. exitiosa* +	**14**
9	9-1	*B. exitiosa* ++ & *B.ostreae* +	**18**
	9-2	*B. exitiosa* ++ & *B.ostreae* +	**1**
